# Modeling Lysosomal Storage Disorders in an Innovative Way: Establishment and Characterization of Stem Cell Lines from Human Exfoliated Deciduous Teeth of Mucopolysaccharidosis Type II Patients

**DOI:** 10.3390/ijms25063546

**Published:** 2024-03-21

**Authors:** Sofia Carvalho, Juliana Inês Santos, Luciana Moreira, Ana Joana Duarte, Paulo Gaspar, Hugo Rocha, Marisa Encarnação, Diogo Ribeiro, Matilde Barbosa Almeida, Mariana Gonçalves, Hugo David, Liliana Matos, Olga Amaral, Luísa Diogo, Sara Ferreira, Constança Santos, Esmeralda Martins, Maria João Prata, Luís Pereira de Almeida, Sandra Alves, Maria Francisca Coutinho

**Affiliations:** 1 Research and Development Unit, Department of Human Genetics, National Institute of Health Doutor Ricardo Jorge, INSA I.P., Rua Alexandre Herculano, 321, 4000-055 Porto, Portugal; sofia.carvalho@insa.min-saude.pt (S.C.); juliana.santos@insa.min-saude.pt (J.I.S.); luciana.moreira@insa.min-saude.pt (L.M.); ana.duarte@insa.min-saude.pt (A.J.D.); marisa.encarnacao@insa.min-saude.pt (M.E.); diogo.ribeiro@insa.min-saude.pt (D.R.); matilde.almeida@insa.min-saude.pt (M.B.A.); mariana.goncalves@insa.min-saude.pt (M.G.); hugoddavid@hotmail.com (H.D.); liliana.matos@insa.min-saude.pt (L.M.); olga.amaral@insa.min-saude.pt (O.A.); 2 Center for the Study of Animal Science—Institute of Sciences, Technologies and Agro-Environment, CECA-ICETA, University of Porto, Praça Gomes Teixeira, Apartado 55142, 4051-401 Porto, Portugal; 3 Associate Laboratory for Animal and Veterinary Sciences, AL4AnimalS, Faculty of Veterinary Medicine, University of Lisboa, Avenida da Universidade Técnica, 1300-477 Lisboa, Portugal; 4 Faculty of Pharmacy, University of Coimbra, Polo das Ciências da Saúde, Azinhaga de Santa Comba, 3000-548 Coimbra, Portugal; luispa@ff.uc.pt; 5 Biology Department, Faculty of Sciences, University of Porto, Rua do Campo Alegre, 4169-007 Porto, Portugal; mprata@ipatimup.pt; 6 School of Medicine and Biomedical Sciences (ICBAS), Faculty of Porto, Rua de Jorge Viterbo Ferreira 228, 4050-313 Porto, Portugal; 7 Newborn Screening, Metabolism and Genetics Unit, Department of Human Genetics, National Institute of Health Doutor Ricardo Jorge, INSA I.P., Rua Alexandre Herculano, 321, 4000-055 Porto, Portugal; paulo.gaspar@insa.min-saude.pt (P.G.); hugo.rocha@insa.min-saude.pt (H.R.); 8 Department of Medical Sciences, Campus Universitário de Santiago, Edifício da Saúde, Agra do Crasto, 3810-193 Aveiro, Portugal; 9 Centre for the Research and Technology of Agro-Environmental and Biological Sciences, CITAB, Inov4Agro, University of Trás-os-Montes and Alto Douro, 5000-801 Vila Real, Portugal; 10 Centro de Referência de Doenças Hereditárias do Metabolismo do Centro Hospitalar Universitário de Coimbra, CR-DHM (CHUC), Praceta Professor Mota Pinto, 3004-561 Coimbra, Portugal; ld@chuc.min-saude.pt (L.D.); saralopesferreira@chuc.min-saude.pt (S.F.); 12944@chuc.min-saude.pt (C.S.); 11 Centro Hospitalar Universitário do Porto, Hospital de Santo António, CHPorto, Largo do Prof. Abel Salazar, 4099-001 Porto, Portugal; esmeralda.martins@chporto.min-saude.pt; 12 Health Research and Innovation Institute, University of Porto, i3S, Rua Alfredo Allen 208, 4200-135 Porto, Portugal

**Keywords:** mucopolysaccharidosis type II, disease modeling, in vitro models, induced pluripotent stem cells (iPSCs), dental pulp stem cells (DPSCs), stem cells from human exfoliated deciduous teeth (SHEDs)

## Abstract

Among the many lysosomal storage disorders (LSDs) that would benefit from the establishment of novel cell models, either patient-derived or genetically engineered, is mucopolysaccharidosis type II (MPS II). Here, we present our results on the establishment and characterization of two MPS II patient-derived stem cell line(s) from deciduous baby teeth. To the best of our knowledge, this is the first time a stem cell population has been isolated from LSD patient samples obtained from the dental pulp. Taking into account our results on the molecular and biochemical characterization of those cells and the fact that they exhibit visible and measurable disease phenotypes, we consider these cells may qualify as a valuable disease model, which may be useful for both pathophysiological assessments and in vitro screenings. Ultimately, we believe that patient-derived dental pulp stem cells (DPSCs), particularly those isolated from human exfoliated deciduous teeth (SHEDs), may represent a feasible alternative to induced pluripotent stem cells (iPSCs) in many labs with standard cell culture conditions and limited (human and economic) resources.

## 1. Introduction

Mucopolysaccharidosis type II (MPS II; #MIM 309900; #ORPHA 580), also known as Hunter syndrome, is a rare genetic disorder that is inherited as an X-linked trait, with an incidence ranging from 0.38 to 1.09 per 100,000 live male births (reviewed in [1]). This disorder belongs to the group of lysosomal storage disorders (LSDs) and is caused by a deficiency in the lysosomal enzyme iduronate 2-sulfatase (IDS; EC 3.1.6.13), which catalyzes the hydrolysis of 2-sulfate groups of dermatan sulfate (DS) and heparan sulfate (HS). Therefore, its deficit causes the pathological accumulation of these two glycosaminoglycans (GAGs), which translates into a multisystemic disease also affecting the brain in at least two thirds of cases (reviewed in [2]).

As many other LSDs, this disorder was first described more than 100 years ago, in 1917, by the Canadian physician Charles Hunter, from whom it got its most colloquial designation [3]. Since then, numerous advances have been made regarding our understanding of this rare disease. It was shown to be a progressive and multi-systemic pathology, and its major causes were disclosed, both at biochemical [4] and molecular levels [5,6], with the disease mapping to the *IDS* gene (HGNC ID: 5389) on chromosome X, which encodes for the lysosomal enzyme previously mentioned. At a genetic level, for example, we now know that Hunter syndrome is characterized by a significant heterogeneity, as no highly recurring mutations have been reported so far, even though some variants seem to be slightly more frequent (reviewed in [7]). Also at the clinical level, much has been discovered since the disorder was first reported. Indeed, what was once thought to be a single disease with two quite divergent clinical presentations, either severe or attenuated (depending on the length of survival and presence/absence of neurological disease), is now known to be a continuum between the two forms, with disease severity linked to relative levels of IDS enzyme (reviewed in [8]). In general, MPS II clinical signs and symptoms include coarse facial features, skeletal deformities and joint stiffness, growth retardation, organomegaly, and significant respiratory and cardiac impairments [9]. Neurological involvement has also been reported in at least two thirds of cases [10,11,12]. Patients also present ENT (ear, nose, and throat) manifestations, sleep disturbances and obstructive apnea [13]. Visual symptoms may also be prominent [14]. According to the natural history of the disease, death occurs typically before adulthood for the most severe forms, while patients suffering from milder forms may usually survive until later in adult life [12] (reviewed in [2]). Also at a subcellular level, much has been learnt on the disease pathophysiology. Increased accumulation of DS and HS was shown to impair numerous cellular functions, including cell adhesion, endocytosis, intracellular trafficking, and intracellular ionic balance. It was also demonstrated to promote nitric oxide synthesis and trigger an inflammatory cascade, with numerous deleterious effects [15]. However, a full characterization of this cascade of secondary cellular events is still lacking (reviewed in [15]).

Currently, the standard of care for MPS II patients is enzyme replacement therapy (ERT), i.e., the intravenous administration of a functional recombinant version of the deficient enzyme. ERT has been shown to reduce urinary GAG levels and liver and spleen volumes in MPS II patients [16] (reviewed in [8]), with real-world data further suggesting that therapy may also improve other somatic cardiorespiratory parameters [17] (reviewed in [8]). Still, it does hold a number of limitations, namely, its inability to cross the blood–brain barrier and act over the neurological symptoms, an issue which is common to virtually all ERT formulations developed so far, regardless of the LSD they apply to. To overcome the limitations of the ERT approach, some modifications to the traditional ERT protocol have actually been tested, including changes in the administration route, the introduction of modified fusion proteins, and the use of alternative hosts for enzyme production (reviewed in [2]). However, some challenges do persist, such as the treatment’s inability to act over the neurological symptoms, its high cost, and life-long dependence [18].

Altogether, the need for deeper understanding of the pathophysiological mechanisms that underlie the disorder and for more effective treatments to counteract it justify the need for a cellular in vitro model that accurately recapitulates the disease phenotype in hard-to-reach/hard-to-treat cells, e.g., those derived from the nervous and/or skeletal systems. Regarding neurons in particular, the use of induced pluripotent stem cells (iPSCs) to model neurogenetic disorders is well established, for example, the function of cortical neurons from patient-derived fibroblasts or blood cells is now well documented and numerous studies in MPS II-derived iPSCs have already been published, with remarkable results and insights on the neuropathology of this disorder [19,20,21] (reviewed in [22]). However, there are notable drawbacks to using these de-differentiated, reprogrammed cells as in vitro models for molecular studies, mostly their high cost and time-consuming technology validation. That is why we are working with a completely different patient-derived cellular model: an alternative, less invasive and less laborious, source of stem cells, the so-called dental mesenchymal stem cells (DMSCs), which can be isolated from different sources in the oral cavity (reviewed in [22]).

Here, we report the establishment of two independent MPS II-derived cultures of stem cells from human exfoliated deciduous teeth (SHEDs) and their subsequent characterization at molecular, biochemical and pathophysiological levels. The existence of this small population of dental pulp stem cells (DPSC) was first reported by Miura and co-workers in 2003 [23], when SHEDs were first isolated from primary teeth that were lost due to the eruption of permanent teeth. That initial paper already provided remarkable insights into this particular population of DMSCs, demonstrating their high proliferative capacity and their ability to differentiate into a variety of cell types, including neural cells, adipocytes, and odontoblasts. Those authors also attempted their in vivo transplantation, showing that SHEDs were able to induce bone formation, generate dentin, and survive in mouse brain [23]. Thanks to the efforts of many independent teams working in different fields, these highly proliferative cells are now fully characterized and their mesenchymal stem cell (MSC) expression profile disclosed ([24,25,26], reviewed in [27]). Soon enough, this DMSC population was considered to hold great promise for regenerative medicine approaches and other cell-based therapies. Indeed, numerous studies have shown that autologous transplantation of SHEDs, for example, may be a safe and promising approach for both dentin and pulp regeneration (reviewed in [28]), but their applications far exceed the orthodontic field, as SHEDs have also been tested for their regenerative potential against spinal cord injury [29,30,31], hypoxic–ischemic brain injury [32], ovariectomy-induced osteoporosis [33], liver fibrosis [34], and systemic lupus erythematosus [35]. And that potential lies not only in the cells themselves but also in the conditioned media one may collect from their culture, as elegantly shown by Katahira and co-workers in a recent study, where they immortalized a SHED cell line and analyzed the effects of its conditioned medium on cutaneous pressure ulcers [36].

In the LSD field, however, DMSCs (and SHEDs in particular) are mostly unknown and their potential unexplored. As such, here we addressed the question of whether these cells could be used, not for therapeutic purposes, but for disease modeling, as they are well known to retain the ability to differentiate into several cell lineages that represent disease-relevant cell types, not only for MPS II, but for LSDs in general (e.g., chondrocytes, osteoblasts, and neuronal cell types; reviewed in [37]). To the best of our knowledge, this was the first time that SHEDs were isolated from LSD patients.

Briefly, we have not only successfully confirmed the DMSC identity of the established MPS II SHEDs but also assessed their LSD phenotype. Regarding their stemness potential, the MPS II-derived SHED cell lines described here presented an expression pattern characteristic of an MSC line, with high expression levels of *CD105*, *CD73* and *CD90* and a weak but still detectable expression of the pluripotency genes *Nanog*, *Oct3-4* and *Sox2*. They were also able to differentiate into endoderm, ectoderm and mesoderm. Also noteworthy, our results so far clearly demonstrate that the typical MPS subcellular phenotype is already present in the established MPS II-derived SHED cell lines. We have also briefly tackled whether secondary accumulation of lysolipids can be detected in those lines using a biomarker panel approach. Overall, this is an original contribution to the LSD field, as it demonstrates the existence of an easily accessible, non-invasive source of MSCs that exhibit visible and measurable LSD subcellular phenotypes. Importantly, those can be collected from severely affected individuals (those who suffer from infantile forms of these disorders). It seems most valuable to use these methods in order to better establish patient-specific models to understand the cellular dynamics of the disease in the donor patients of such cells.

## 2. Results

### 2.1. Establishment of Primary SHED Cell Cultures from Patients and Controls

In this study, two unconventional MPS II samples were used to establish novel patient-derived MSC lines in house: SHEDs. The teeth included in the study were non-carious, had no previous restorations, and had no reports of prior trauma, even though one was surgically extracted (that from patient MPS II, 2.01). All other samples were spontaneously exfoliated teeth (namely, those obtained from patients MPS II, 2.02 and controls 01 and 02, here termed Ctrl.01 and Ctrl.02).

**Figure 1:**
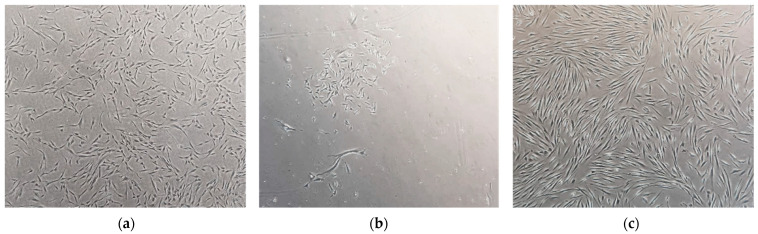
Optical microscope images (4×) of an MPS II-derived SHED primary culture. Undifferentiated SHEDs have a spindle shape similar to fibroblasts. (**a**) Fusiform format; (**b**) small colony formation (CFU-F); (**c**) sub-confluence. Cells were observed using a Leica DMIL inverted contrasting microscope (Leica Microsystems, Wetzlar, Germany) with 4× magnification.

Upon reception of the biological sample, dental pulp was extracted, enzymatically digested, and left to grow in poly-D-lysine or vitronectin-coated 12-well plates. Cells derived from the dental pulp were visible within 1–2 weeks. Then, in a process that took anywhere from two weeks to one month, we could observe a population of SHEDs, morphologically characterized by spindle-shape cells, similar to fibroblasts (Figure 1a), which initially formed small colonies (Figure 1b) that were left to grow until they reached sub-confluence (Figure 1c). Throughout the whole process, which involved the establishment of the primary cultures, their passage, freezing and thawing, SHED cell viability and morphology were checked using a light microscope and any relevant alteration noted. All established cell lines shared the same morphological features, regardless of whether they were derived from patients or controls.

### 2.2. The Established SHED Cell Lines Share an MSC Phenotype Identity

The MSC phenotype of the established SHED cultures was assessed through different protocols, namely, quantitative real-time reverse-transcription polymerase chain reaction (qRT-PCR) analysis of different stem cell markers and multi-lineage differentiation assays.

Regarding the qRT-PCR analysis, total RNA was successfully extracted not only from cultured SHEDs but also from an iPSC line already available in the lab (INSAi002-A; derived from Fabry fibroblasts [38]). The extracted RNA samples were reverse-transcribed into cDNA, and specific gene expression was assessed (Table 1). In summary, *CD34* was not detected as expected for SHEDs. *CD105*, *CD73* and *CD90* were highly expressed and *CD166*, *MHC I* and *CD117* showed strong to moderate expression. The pluripotency genes *Nanog*, *Oct3-4* and *Sox2* were also expressed, although weakly (Ct value > 35). Moreover, weak expression of *MHC II* was also detected in SHEDs. The expression levels observed for the iPSC line are also summarized in Table 1. Briefly, INSAi002-A iPSCs displayed high expression levels of *CD105*, *CD73*, *CD90*, and *Oct-3/4* and moderate expression levels of *MHC II*, *Nanog*, and *Sox-2*. Absolute ΔCt valuescalculated using glyceraldehyde 3-phosphate dehydrogenase (*GAPDH)* and β-actin *(ACTB)* as housekeeping genes are listed in Table S1.

**Figure 2:**
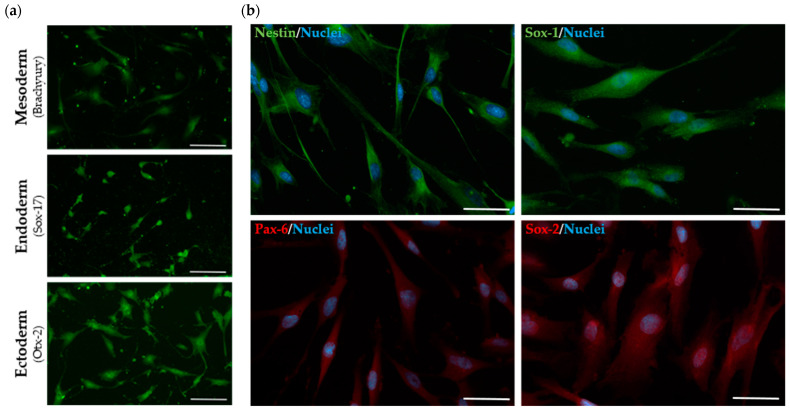
Immunostaining of MPS II-derived SHEDs. (**a**) Confirmation of 3-germ-layer differentiation capacity: mesoderm (top), endoderm (middle), ectoderm (down). (**b**) Confirmation of neural progenitor cell (NPC) stage of SHEDs: Nestin and Sox-1 (top line); Pax-6 and Sox-2 (bottom line). Scale bar: 50 µm. Images were acquired using a DM400 M fluorescence microscope (Leica, Wetzlar, Germany).

Further insights on the stem nature of the established SHED cell lines came from multi-lineage differentiation assays using a human pluripotent stem cell functional identification kit (R&D Systems, Minneapolis, MN, USA). This kit contains especially formulated media supplements and growth factors that can be used to differentiate human pluripotent stem cells into endoderm, ectoderm and mesoderm. It is a fast protocol, which relies on 2- to 4-day incubations with those media, and also includes antibodies to characterize each of the three primordial germ layers: Sox17 for endoderm; Otx2 for ectoderm; and Brachyury for mesoderm. Not surprisingly, all established cell lines stained positive for each respective marker, thus confirming their capacity to differentiate into each of the three germ layers (Figure 2a).

### 2.3. The Established SHED Cell Lines Express Major NPC Markers

As a final characterization step of the established SHED cell lines, the early DMSC commitment to their so-called “neuronal fate” was also confirmed. This study was performed in primary SHED cells before any neuronal differentiation protocol was attempted, and relied on the use of a commercially available kit. Briefly, their neural progenitor cell (NPC) stage was analyzed based on the expression of major NPC markers (Nestin, Sox-1, Sox-2 and Pax-6), with a positive fluorescence pattern observed for all four markers evaluated (Figure 2b). No significant differences were detected between healthy and MPS II cell lines in early passages (from passage 5 to passage 7).

### 2.4. The Established MPS II-Derived SHED Cell Lines Display Disease-Related Biochemical and Molecular IDS Defects

Whenever an MPS tooth was received in the laboratory, the only information available was the type of MPS from which the patient it belonged suffered. Therefore, as soon as its derived SHED cell line was established and the first vials stored, cell pellets were collected and used for mutational analyses and enzyme activity assays.

#### 2.4.1. The Presence of Pathogenic *IDS* Variants Was Confirmed in the Established MPS II Patient-Derived SHED Cell Lines

For molecular analyses, DNA was successfully extracted from both MPS II patient-derived SHED cultures, and standard amplification and Sanger sequencing protocols were employed to analyze the nine *IDS* exons plus their surrounding intronic regions, as previously reported [39]. Whenever standard procedures were not enough to identify the disease-causing variant, additional studies were performed, namely, restriction fragment length polymorphism (RFLP) [39] (see Materials and Methods, Section 4.4.1).

Briefly, Case 2.01 was confirmed to carry a rearrangement involving recombination between intron 7 of the *IDS* gene and sequences located distally of exon 3 in the *IDS* pseudogene (*IDS-2*) [GAATC > AGAGG (*IDSP1* > *IDS*)]. This recombination event had already been reported, and is known to cause a partial inversion of the *IDS* gene [40]. Conversely, Case 2.02 was shown to be hemizygous for a variant much more straightforward to detect: the previously reported c.22C > T (p.R8*) nonsense mutation [40]. Both identified genotypes were in accordance with an MPS II phenotype.

#### 2.4.2. IDS Enzyme Activity Is Significantly Decreased in MPS II Patient-Derived SHED Cells

**Figure 3:**
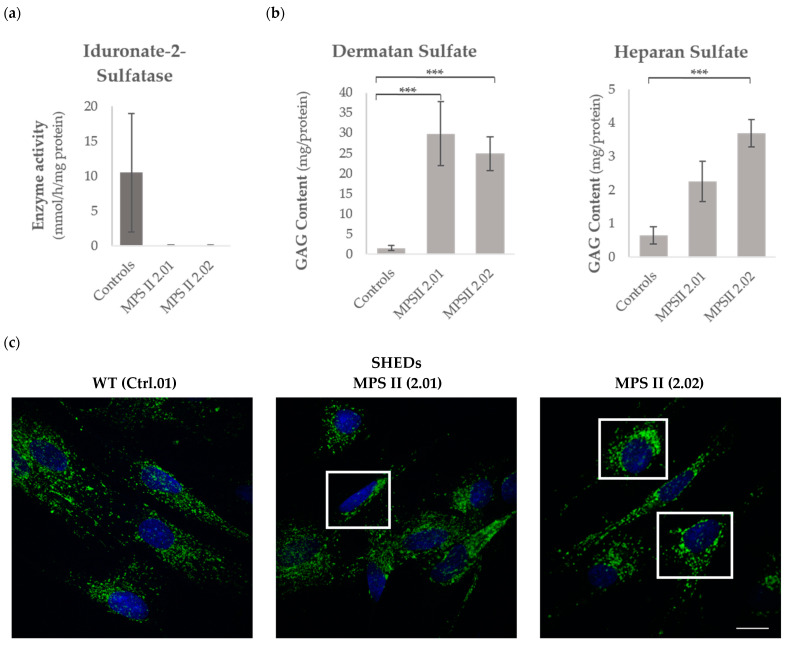
Biochemical profile of SHEDs. (**a**) IDS enzyme activity. Data are presented as means ± SEM (*n* = 3) *** *p* < 0.0005. (**b**) HS and DS levels of control- and MPS II-derived SHEDs. (**c**) Representative confocal images of control (left) and MPS II-derived SHEDs (middle and right) immunostained for lysosomal-associated membrane protein 1 (LAMP1). Scale bar: 25 µm. The outlined regions (white squares) highlight the different lysosomal positioning in controls vs. patient-derived SHEDs, with more pronounced LAMP1 staining in the perinuclear region of MPS II SHEDs. Images were acquired using a TCS-SPE confocal microscope (Leica, Wetzlar, Germany).

When the IDS activity was measured with the fluorescent substrate 4-methylumbelliferyl (4MU)-α-L-iduronate-2-sulfate in SHED cell lysates from both controls and the two independent MPS II patients, no activity could be detected in case 2.01 or 2.02, whilst control Ctrl.01 and Ctrl.02 cells showed normal IDS activity (Figure 3a). The other lysosomal enzymatic assays performed were within the normal range in all the SHED lysates (Supplementary Figure S1).

### 2.5. MPS II Patient-Derived SHED Cells Exhibit a Subcellular LSD Phenotype

Subsequently, the LSD phenotype of the established MPS II patient-derived phenotype was assessed. Briefly, we checked not only the primary GAG storage, which is known to trigger the whole MPS II pathophysiological cascade, but also the lysosomal localization pattern, to check whether it was altered.

#### 2.5.1. GAG Accumulation Is Evident in MPS II Patient-Derived SHED Cells, despite Their High Proliferation Rate

Decreased (or absent) IDS activity in MPS II patients is known to cause an intracellular and extracellular accumulation of two major GAGs, namely, HS and DS. Thus, the levels of those two GAGs were measured by liquid chromatography–mass spectrometry/mass spectrometry (LC-MS/MS), and both MPS II SHED cultures showed evident GAG accumulation when compared with control SHEDs (Figure 3b).

#### 2.5.2. LAMP1 Staining Is Altered in Patient-Derived SHED Cells

Accumulation of DS and HS causes lysosomal hypertrophy and an increase in the number of lysosomes in cells. Therefore, any method that allows for an evaluation of the number, size and sub-cellular localization of these disease-relevant organelles may provide relevant data on the health/disease status of a given cell line. Here, we chose to perform a lysosomal-associated membrane protein 1 (LAMP1) immunostaining protocol in control vs. MPS II cells. LAMP1 is one of the most abundant lysosomal membrane proteins, nicely correlating with lysosomal dysfunction, as its overexpression reflects abnormal accumulation of lysosomes. Overall, the pathological phenotype was quite evident, with MPS II cells presenting a prominent LAMP1-positive perinuclear fluorescence when compared to controls (Figure 3c).

### 2.6. MPS II Patient-Derived SHED Cells Present Normal Lysosphingolipid Levels

Apart from the primary accumulation of GAGs (assessed in Section 2.5.1), secondary storage of lipids is also known to play a role in the pathophysiological cascade that gives rise to MPS symptoms. Here, we used a multiplexed LC-MS/MS method to simultaneously quantify four different glycosphingolipid biomarkers [Lyso-Gb3, Glc-Sph, Lyso-SM, and PPCS (initially referred to as Lyso-SM-509)] in our MPS II SHEDs vs. controls, and observed normal levels of all four lysosphingolipids in the samples analyzed (Table S2).

## 3. Discussion

Here, we describe the establishment and characterization of two MPS II patient-derived SHED cell lines (named 2020TF-MPS2.01 and 2020TF-MPS2.02) while addressing their modeling potential for this severe condition by evaluating whether they display visible and measurable MPS subcellular features.

Briefly, we successfully implemented a protocol developed by Goorha and Reiter [41] for efficacious remote tooth collection and subsequent dental pulp extraction for growth and expansion of that particular subset of DMSCs, and applied it in two independent MPS II cases and a significant number of controls (>30). As originally reported by Goorha and Reiter, the process of growing these particular DMSCs can take anywhere from 1 to 2 weeks, and—at least in our hands—there seems to be no particular correlation between the size of the pulp or the time it takes to arrive at the lab (as long as the 48/72 h interval is ensured) and the time it takes for the first cells/colonies to become visible in the plate. Overall, the whole method is well described in the publication we refer to, and it is not hard to implement in a lab with standard cell culture conditions, regardless of whether the operators have had experience with other sorts of stem cells, namely, iPSCs.

Technically, MSCs are classified as multipotent stem cells and not as pluripotent stem cells. Still, as we have already seen, they do present a positive expression pattern of *Oct-3/4*, *Nanog*, and *Sox-2*, which are standard pluripotency markers [42,43,44]. DMSCs in general and SHEDs in particular have been known to express those markers for quite a while. In fact, that characteristic was already reported in healthy SHEDs back in 2006 in an original paper by Kerkis et al. [24], where their stemness character was confirmed [25]. Nevertheless, the expression level of any of these markers when compared with other commonly assessed MSCs markers is known to be weak. These data correlate nicely with our results, where all SHED cell lines presented with positive expression levels of these three pluripotency markers, but at a level that was significantly lower than that seen for specific MSC markers (*CD105*, *CD90*, and *CD73*). They also seem to be in accordance with what we saw on the iPSC cell line we used as a control (INSAi002-A; derived from Fabry fibroblasts [38]): while positive, the levels of expression of *Oct-3/4*, *Nanog*, and *Sox-2* were much lower in the established multipotent SHED cell lines than in the truly pluripotent iPSC line, which was triggered to overexpress those markers through a reprogramming protocol with Yamanaka factors. Remarkably, no studies comparing the expression levels of stemness markers between DMSCs and iPSCs are available, at least that we are aware of. Therefore, these results become even more interesting.

Specific MSC markers were also measured in both healthy and disease-derived SHEDs, as well as in the iPSC line INSAi002-A, and overall, the results were in line with what would be expected according to the literature: MSC markers (*CD105*, *CD90*, and *CD73*) were the ones that displayed higher expression levels, thus supporting the MSC phenotype of the established SHEDs. The two remaining markers assessed, *CD34* and *MHC II*, are commonly described as absent in MSCs [45,46]. They did, however, show positive expression, even though with significantly lower levels than those observed for *CD105*, *CD90*, and *CD73*. They were actually comparable with the ΔCt value observed for the pluripotency markers. While this result seems unaligned with MSC requirements, as they are reported in the bibliography, when we look at individual papers where SHED and DPSC expression patterns for these markers were assessed, this observation is actually common. For example, recently, positive expression levels of *MHC II* were reported in a commercially available DPSC line and considered a normal aspect [47]. Additionally, there is already literature commenting on the possibility that the absence of expression of those markers may not be mandatory for a cell to be classified as MSC, once several MSCs have been shown to express, at least to some extent, both of them [45,46].

Adding to our qRT-PCR results regarding the MSC phenotype are our results on the differentiation capacity of the established SHED cell lines into distinct cell types. Traditionally, one of the listed requirements to identify MSCs is their ability to differentiate into three different cell types: adipocytes, osteocytes and chondrocytes [48]. More recently, though, many authors have argued those requirements should be updated to include cells from the three germ layers ectoderm, mesoderm, and endoderm [49]. That is why we chose to perform a novel (and faster) 4-day-long tri-lineage differentiation protocol, through which our MPS II patient-derived SHED cell lines were forced to differentiate into ectoderm, endoderm and mesoderm. Overall, the protocol worked precisely as anticipated, with the cells staining positive for all tested markers, regardless of the differentiation attempted.

Still, there was yet another assessment we considered to be relevant regarding the overall nature of the established cell lines, regardless of their health/disease status. SHEDs have a behavior similar to neuronal precursor cells. Indeed, there are now numerous publications providing evidence that SHEDs express neuronal and glial cell markers, owing to the neural crest-cell origin of the dental pulp [50] (reviewed in [51]). And in fact, staining of neuronal markers in SHEDs not subjected to any type of neurodifferentiation protocol, revealed a positive fluorescence pattern for all four markers evaluated—Nestin, Sox-1, Pax-6 and Sox-2—further validating the assumption that SHEDs may actually be classified as NPCs, as stated by several different authors.

Having extensively demonstrated the stemness capacity of all established SHED cell lines and further characterized them as NPCs, we moved on to analyze whether they were able to mimic the primary defect underlying the MPS II phenotype in the patients from whom they were derived. Thus, a careful molecular characterization of their associated genotypes was performed, together with a quantification of each one’s defective enzyme.

Unsurprisingly, when the two established MPS II cell lines were molecularly characterized, both cases were shown to harbor pathogenic *IDS* variants. Case 2.01 was shown to be hemizygous for a complex rearrangement that results from a recombination event between intron 7 of the *IDS* gene and sequences located distally of exon 3 in the *IDS* pseudogene (*IDS-2*) [GAATC > AGAGG (*IDSP1* > *IDS*)] and causes a partial inversion of the *IDS* gene [40]. Case 2.02 was shown to be hemizygous for the previously reported c.22C > T (p.R8*) nonsense mutation [52]. This mutation had already been reported in different populations, correlating either with severe or intermediate forms of the disease [52,53,54]. Altogether, both mutations may easily correlate with severe, early-onset phenotypes, as those presented by both patients included in this study (see Table 2).

Accordingly, when IDS enzyme activity was measured in the lysate of MPS II patients’ SHEDs, it was shown to drop to zero (Figure 3a).

While the results so far already testify to the overall potential of patient-derived SHED cell lines to accurately express a measurable LSD phenotype, the ones we will now focus on further highlight the uniqueness of this cell model.

Our results regarding MPS II-related primary storage in patient-derived SHEDs, for example, are worthy of additional discussion. Briefly, when we quantified DS and HS in healthy and diseased SHED cell lysates by butanolysis derivatization [55], significant differences were observed between patients and controls, with both MPS II samples showing GAG accumulation (Figure 3b). This is particularly relevant when compared with the results other authors have achieved with MPS II iPSC-derived cell lines. Currently, there are numerous reports on the generation of MPS II human iPSC lines. Still, not all papers evaluated the LSD phenotype they present. Instead, most publications focus only on the iPSCs’ generation and characterization protocol, already well established to validate an iPSC line: they report the method of reprogramming, present proof on the established cell line(s’) pluripotency and differentiation potential; assess identity compared to the cells they were reprogrammed from, as well as their karyotype to confirm it remains normal; double-check the presence of the original disease-causing variant, and rule out mycoplasma contamination. There is, however, an original publication by Kobolák and co-workers [20] where numerous analyses were performed, not only in MPS II-derived iPSCs but also, and perhaps most importantly, in NPCs and terminally differentiated (TD) neurons generated from them. Briefly, they used iPSC lines generated from three independent MPS II patients, a healthy control, and a carrier, to generate NPCs and TD neuronal cells, and compared results amongst all those lines. Curiously, all the three MPS II NPC cultures analyzed in that work showed lower total GAG levels (*p* < 0.05) compared to either control or carrier cell lines. For the hallmark accumulation to (finally) be observed, the authors had to promote the terminal differentiation of those MPS II iPSC-derived NPCs into cortical neural cells. Only then could a marked GAG accumulation be detected, though for two of the three MPS II cell lines alone. The third patient-derived MPS II neuronal cell line did not differ significantly from the control cell line [20] (reviewed in [22]). This is significantly different from our own observation in MPS II SHED cell lines, where GAG storage was quite evident. And while we cannot find the reasoning for this discrepant observation, it is (quite) obvious that the results further highlight the disease modeling potential of this simple and easily accessible type of stem cell.

Similar results were obtained regarding LAMP1 staining. Again, the established MPS II SHEDs showed obvious differences compared to the controls, a pattern that is in accordance with previous reports from other teams that observed increased LAMP1 levels in LSD animal models, as well as in human patients. For MPS II in particular, Morimoto and co-workers recently reported increased Lamp1 staining in the brains of MPS II mice. Importantly, when those mice were treated with the recombinant enzyme idursulfase, irrespective of the dosing regimen, intensity decreased in most regions of the brain. Those observations further support the assumption that abnormal Lamp1 staining in MPS II correlates with lysosomal dysfunction [56]. Altered staining patterns for both LAMP1 and LAMP2 were also observed in MPS II iPSC-derived neural stem cells [21]. Remarkably, however, this pattern was not seen in all MPS II-derived neural stem cells reported so far. Indeed, in the original publication by Kobolák et al. [20], to which we have already referred, significant differences between patient- and control-derived cells were only observed in TD neuronal cells, where indeed MPS II samples showed cell-type specific differences in their LAMP2 staining patterns, with many more LAMP2^+^ vacuoles in GFAP^+^ astrocytes than in MAP2^+^ neurons [20]. Again, these significant differences between the novel, naturally occurring stem cell model here reported (patient-derived SHEDs) and its iPSC-derived equivalent (NPCs) further highlight the advantage of establishing this type of cell line.

Finally, we moved on to check whether it was possible to document any downstream effects of the observed primary GAG storage with routine methods. As such, we checked whether a biomarker panel approach for lysosphingolipid quantification could help us obtain an insight into the common downstream pathophysiological mechanism(s) leading to intracellular dysfunction in these disorders. Lysosphingolipids are free sphingoid bases that can be accurately quantified by LC-MS/MS and have great diagnostic value for a number of LSDs characterized by the disturbed catabolism of sphingolipids, the so-called glycosphingolipidoses. Indeed, increased levels of glycolysosphingolipids are present in tissue and plasm of several sphingolipidoses, and some of them have actually been validated as disease biomarkers, namely, globotriaosylsphingosine (Lyso-Gb3) glucosylsphingosine (Glc-Sph) and phosphorylcholine-sphingosine (Lyso-SM) for Fabry, Gaucher and Niemann–Pick type A/B diseases, respectively [57]. However, disruptions of the glycosphingolipid degradation pathways are implicated not only in glycophingolipidoses but also in other LSDs, including MPSs [15]. And it is true that not only there is a well-documented secondary accumulation of lipids in MPS diseases (MPS II included) [21,58] but also there is an increasing body of evidence documenting significant elevations of several glycophingolipidose biomarkers in other LSDs, including neuronopathic forms of MPSs [59]. That is why we tried to address the question of whether any of those biomarkers could be altered in the cell lines under analysis.

While for the two patients here analyzed, no large increases in any of the assessed lysosphingolipids were seen, we do consider it relevant to perform this kind of analysis in other cell models of non-glycosphingolipid diseases, as they may give us an insight into other relevant pathways that can be secondarily impaired. Here, we used exactly the same method that we have implemented in-house for diagnostic purposes in plasma/leukocyte samples. For other labs like ours, with a strong component of diagnostic services, this may be an interesting second-tier test in newly established cell models without requiring additional investment. We are currently recruiting more patients for deciduous tooth collection. In the future, additional analyses will be performed in the established cell lines.

**Figure 4:**
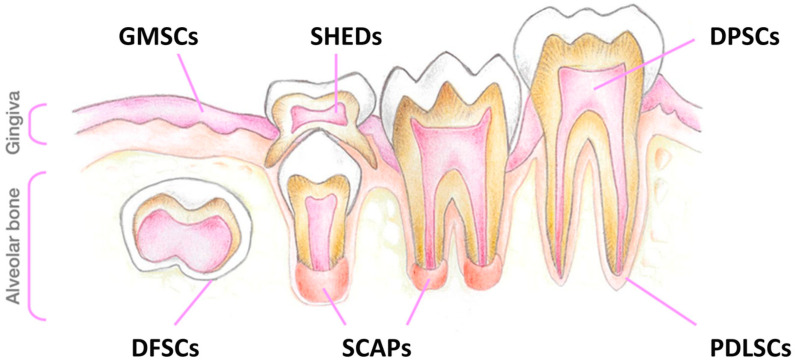
Schematic drawing illustrating the different sources of dental mesenchymal stem cells (DMSCs) in the oral cavity. Abbreviations: GMSCs, gingiva-derived mesenchymal stem cells; DFSCs, dental follicle stem cells; SHEDs, stem cells from exfoliated deciduous teeth; SCAPs, stem cells from the apical papilla; DPSCs, dental pulp stem cells; PDLSCs, periodontal ligament stem cells (reproduced from [22]).

It is also worth reinforcing that there are many other DMSCs, which may be collected from the oral cavity (Figure 4). We have focused our attention on SHEDs, because they may be collected in a non-invasive way in pediatric patients. However, other sources may be considered, particularly for adult patients with milder forms of the disorders, who tend to be diagnosed later in life. A good example is the use of adult human third molar teeth, from where DMSCs may also be isolated. While there are slight variations in the protocols described in the literature for the isolation of DPSCs from this source, the overall method is not significantly different from the one reported here for SHED cell culture.

Overall, this type of sample would allow for a significant increase on the number of eligible patients, because their recruitment platform would be much larger than the current one: it would move from children who are currently losing their baby teeth to virtually any patient, regardless of his/her age. The fact that the surgical removal of human third molars (also known as wisdom teeth) is the most common surgical procedure in the orthodontic field, which also adds to the interest in implementing this protocol and asking for this type of sample. This picture is probably even more prominent in individuals who suffer from MPS, particularly from the skeletal forms of these disorders. In fact, some of the most common and obvious orofacial abnormalities in MPS patients are maxillomandibular abnormalities. GAG accumulation in soft tissues, cartilage, and bones and secondary cellular responses to accumulated GAGs are probably the culprit for abnormalities in orofacial soft tissues, orofacial bones, and teeth [60]. That is why MPS patients are frequently subjected to tooth-removal surgeries, among other orofacial interventions.

Finally, it is also worth mentioning that while we recognize the therapeutic potential any stem cell line may eventually hold, particularly in a field where hematopoietic stem cell transplantation (HSCT) is a feasible and recommended approach for a few disorders, depending on the severity of the phenotype and the age of the patient, our goal with this work was never to either establish or characterize SHED cell lines for therapeutic purposes. The cells we isolated present the same genetic defect harbored by their donor; therefore, only after gene editing (e.g., CRISPR-Cas system) would they be suitable for transplantation. Plus, as these cells are isolated from naturally exfoliated baby teeth, one would only have access to that sample when these patients started losing their teeth, i.e., at about age 6 or later. And that is certainly late for HSCT approaches, as it is well documented for other MPS [namely, MPS I with central nervous system (CNS) affection] that HSCT only works in children who are less than 2.5 years of age. Additionally, it is also worth mentioning that the protocol we used here is not suitable to establish clinical-grade stem cells.

Altogether, our results show that this patient-derived sample is a much faster and more economical way to establish a stem cell model, and it also holds the potential to display disease-relevant sub-cellular features. Thus, patient-derived SHEDs may be assessed not only to allow a better understanding of the pathophysiological mechanisms underlying the disorder but also to evaluate the potential impact of novel therapeutic approaches in vitro.

## 4. Materials and Methods

### 4.1. Materials

Methanol, acetonitrile, chloroform, and ethanol were from Fisher Chemicals. Formic acid and DMSO were from Sigma-Aldrich (Gillingham, UK). All solvents were HPLC-grade. Water used was Milli-Q grade (Milipore, UK). Antibodies used in this study are listed in Table 3. All other specific reagents and materials are listed in the following subsections.

### 4.2. Patients and Samples

#### 4.2.1. Patient 1 (Case MPS 2.01)

Patient 1 was the first child of a non-consanguineous couple. While there was no access to a complete family tree, family history records included a reference to the mother’s learning difficulties and another to a disabled maternal cousin (son of a maternal great aunt), who died in childhood without diagnosis.

At 22 months, initial signs of developmental delay, mostly regarding language, were reported, accompanied by other clinical features, which included wide-based gait with knee flexion and motor agitation. It was also at this age that a first reference was made to a “rough face” in the records of an objective examination of pediatric consultations.

By then, the patient’s skeletal and somatic features included coarse facies, stiff joints and postnatal macroglossia. Some cardiac manifestations were also detected, namely, interventricular communication (IVC) and patent ductus arteriosus (PDA), both solved by now. At 35 months, thickening of the aortic valve was detected. Additionally, moderate aortic insufficiency and left ventricular hypertrophy were also referenced. ENT manifestations were also present, including chronic nasal obstruction, even though no recurrent otitis or hearing deficit have been detected so far. Finally, mild psychomotor development retardation was also reported.

Upon objective examination at the metabolic and genetic consultation (also at 35 months of age), the child displayed relatively understandable language and showed important emotional lability. His dysmorphic features (namely, coarse face, abundant and thickened eyebrows, macroglossia apparent, abnormal posture with knee flexion and wide-based gait and limitation in elbow extension, slight pectus carinatum with far apart nipples and preputial ring punctiform) were considered suggestive of MPS and the child was referred for molecular and biochemical diagnosis. There was no evidence of hepatosplenomegaly or hernias. Regarding evolution of weight gain and head circumference, there was a positive crossing of percentiles of the various parameters: height at percentile (P) 85–97, weight at P > 97 [with body mass index (BMI) at P > 99, which corresponds to a Z-score > 3SD (where SD stands for standard deviation of the reference population)] and head circumference at P > 97.

#### 4.2.2. Patient 2 (Case MPS 2.02)

Patient 2 is a 6-year old male child, diagnosed at 2 years old with MPS type II. The child presented with multisystemic involvement, which included not only several classical musculoskeletal features of the disease (inguinal hernias, claw hands and low stature) but also a series of other symptoms, which included hypertrichosis, hepatomegaly and cardiac involvement (minimal mitral regurgitation). Later, he underwent otorhinolaryngological surgery to perform bilateral myringotomy and tubes with adenoidectomy. Currently, his inguinal hernias have already corrected. Recently (June 2022), his global psychomotor development was assessed and considered normal. Nevertheless, he developed an orthopedic condition with joint restrictions, which requires physiotherapy.

#### 4.2.3. Biological Samples

Deciduous teeth (i.e., “baby” teeth) from two independent MPS II patients and from an equal number of controls were acquired.

Briefly, the process of sample collection was performed as follows: all subjects included in this study, either wild-type (WT) controls or MPS II patients, received a tooth collection kit, which included a parafilm-sealed Falcon tube filled with adequate transport media accompanied by return instructions, a biohazard bag, plus a prefilled delivery form. Also included in the kit was an informed consent form to be completed by the participant’s legal representative, a summary of the project and its objectives, and a flyer with major recommendations and frequently asked questions (See Figure S2). The families were instructed to store the Falcon tube in the refrigerator (4 °C) and to place the tooth in it, virtually as soon as it fell (within 20 min), and send it to the lab within 24 h.

While we received more than 50 deciduous teeth and established over 30 independent control cell lines from them, most of the results here reported were obtained through the analyses of only two of those controls (here termed Ctrl.01 and Ctrl.02). An exception was made for enzyme activity and LC-MS/MS assays, where a significantly higher number of controls was included, to account for interindividual differences.

In general, the deciduous teeth used in this work were spontaneously exfoliated teeth. However, for patient 1 (MPS II, 2.01), a surgically extracted deciduous tooth was received.

### 4.3. Cell Cultures

#### 4.3.1. Establishment of Primary SHED Cell Cultures

The whole protocol for the establishment of primary SHED cell cultures was derived from that published by Goorha and Reiter in 2017 [41] for remote tooth collection of exfoliated teeth and subsequent production of DPSC cultures for differentiation or storage, with minor alterations. In detail, upon every tooth reception, and after a careful inspection of the transport medium to discard possible microbial contamination, teeth were broken open under sterile conditions, using a sterilized hammer. The dental pulp was then pulled out and placed into preheated washing media [saline solution, with PenStrep (50 U/mL) fungizone (1.25 µg/mL)] in a small petri dish, where it was minced into smaller fragments. After a brief centrifugation, the minced pulp was resuspended in fresh DPSC culture medium [DMEM/F12 (1:1) with 20% FBS, PenStrep (50 U/mL) and fungizone (50 U/mL)] containing 3 mg/mL collagenase (Gibco, Thermo Fisher Scientific, Waltham, MA, USA) and 1–4 mg/mL dispase (Neutral Protease Grade II, Roche, Basel, Switzerland) and incubated for 1 h at 37 °C. Following enzymatic digestion, samples were centrifuged at 448× *g* during 5 min, resuspended in 1 mL of DPSC culture medium and seeded in a single well of a poly-D-lysine- (Gibco, Thermo Fisher Scientific, Waltham, MA, USA) or vitronectin (Thermo Fisher Scientific, Waltham, MA, USA)-coated 12-well tissue culture plate. The plate was then left to incubate overnight in a standard 37 °C incubator, with 5% CO_2_. The following day, supernatant was removed from the well and briefly centrifuged to spin down the floating, unattached cells. The resulting pellet was resuspended in fresh DPSC culture media and seeded in another coated well from the same plate. Cultures were washed with the previously described washing solution and media changed twice a week, up until cells reached sub-confluence (80–90%).

#### 4.3.2. Passage, Freezing and Thawing of SHED Cell Cultures

Again, in accordance with the original protocol developed by Goorha and Reiter [41], SHED cultures were passaged at a ratio of 1:3, according to the following rationale: one part was sub-cultured for passage, and the remaining two were frozen for future applications. In higher passages, when there was no need to assure the maximum possible number of stored vials to ensure the preservation of the cell line, parts 2 and 3 were frequently used to generate pellets for subsequent analyses. For cell detachment, Accutase (GRiSP, Lda., Porto, Portugal) was used. Early-passage cells were frozen for long-term storage in liquid nitrogen, in culture media supplemented with 15% DMSO (Sigma-Aldrich, St. Louis, MO, USA), according to standard freezing protocols.

### 4.4. Molecular Analyses

#### 4.4.1. Genotype Assessment by PCR and Sanger Sequencing

Genomic DNA was automatically extracted and purified from cell pellets on a BioRobot EZ1 instrument (QIAGEN, Germantown, MD, USA) using the EZ1 DNA Tissue Kit (QIAGEN, Germantown, MD, USA). Previously reported primers [39] covering all *IDS* exons and their surrounding intronic regions, as well as its promoter region, were used to sequence the gene of interest. Each PCR reaction was carried out using approximately 40 ng of genomic DNA, 1X the PCR reaction mix ImmoMix™ Red (Bioline, London, UK) and 0.25 µM of each primer. For some particular fragments, betaine and/or DMSO were also used to enhance the PCR amplification of the target region (conditions available upon request). Then, samples were heated to 95 °C for 7 min, followed by 35 cycles of denaturation, annealing and extension. The final extension was completed by 5 min at 72 °C. The amplified fragments were purified with illustra ExoStar™ 1-Step (GE Healthcare Life Sciences, Buckinghamshire, UK) and sequenced using a BigDye Terminator v1.1 Cycle Sequencing Kit (Applied Biosystems, Foster City, CA, USA) on an ABI PRISM 3130xl Genetic Analyser (Applied Biosystems, Foster City, CA, USA). Results were analyzed with FINCHTV sequence analysis software, version 1.3.1. Sequencing profiles were compared with the *IDS* reference sequence ENST00000340855.11 [https://www.ensembl.org (accessed on 1 December 2023)] using the Clustal Omega multiple sequence alignment bioinformatic tool [https://www.ebi.ac.uk/Tools/msa/clustalo/ (accessed on 1 December 2023)]. The presence of the variants found in both patients was also confirmed at cDNA level. Briefly, total RNA was extracted from independent cell pellets using GRS Total RNA-Blood & Cultured Cells Kit (GRiSP, Lda., Porto, Portugal). 1 µg of RNA was reverse-transcribed using the Ready-To-Go You-Prime First-Strand Beads kit (GE Healthcare Life Sciences, Chicago, IL, USA), according to the manufacturer’s instructions.

Importantly, only after fully genotyping the patients were the results confirmed by the clinicians, who had access to the patients’ clinical file and molecular results from previous analyses.

#### 4.4.2. qRT-PCR Analyses

qRT-PCR was performed for MSCs’ related gene sequences from Bio-Rad^®^ (Bio-Rad Laboratories, Hercules, CA, USA) to confirm the DPSC/MSC phenotype identity of the established cell lines: *CD34* (qHsaCID0007456), *CD90/THY1* (qHsaCED0036661), *CD73/NT5E* (qHsaCID0036556), *CD105/ENG* (qHsaCID0010800), *CD166/ALCAM* (qHsaCID0037887), *CD117/c-kit* (qHsaCID0008692), *Sox2* (qHsaCED0036871), *Oct-3/4/POU5F1* (qHsaCED0038334), *MHC I/HLA-A* (qHsaCED0037388), *MHC II/HLA-DRA* (qHsaCED0037296) and housekeeping genes: *ACTB* (qHsaCED0036269), *GAPDH* (qHsaCED0038674).

Total RNA was extracted as described in the previous section, and reverse-transcribed using the same kits. Nevertheless, for qRT-PCR analyses, lower amounts of total RNA were used for cDNA synthesis (0.5–1 µg). qPCR was performed in a Bio-Rad CFX 96 Touch Real-Time PCR Detection System (Bio-Rad Laboratories, Hercules, CA, USA) apparatus using the iTAQTM SYBR^®^ Green Supermix (Bio-Rad Laboratories, Hercules, CA, USA). All plates were designed to contain duplicates of targeted human genes as well as a negative control. Recommended Bio-Rad PrimePCR cycling protocol was employed in all cases: 95 °C for 2 min (activation), 40 cycles comprising 95 °C for 5 s (denaturation), 60 °C for 30 s (annealing), and 65–95 °C (0.5 °C increment), 5 s/step (melt curve). The number of cycles for each well was recorded. Data were processed using Bio-Rad CFX^®^ Manager Software 3.1 (Bio-Rad Laboratories, Hercules, CA, USA). Fold differences were calculated using the standard ΔCq method with *GAPDH* and *ACTB* as housekeeping genes.

### 4.5. Biochemical Analyses

#### 4.5.1. Fluorometric IDS Enzyme Assay

Control (WT) and MPS II-derived SHEDs were used in the determination of IDS enzyme activity. Briefly, cell homogenates were prepared by sonication, and their protein concentration determined using the Pierce™ BCA Protein Assay Kit (TermoFisher Scientific, Waltham, MA, USA) and measured by spectrophotometer (VICTOR^®^ Nivo^TM^ Plate Reader, PerkinElmer Inc., Waltham, MA, USA). Then, the IDS enzyme activity was assayed using fluorogenic substrate 4MU-α-L-iduronate-2-sulfate, according to the method described by Voznyi et al. [61], and the fluorescence was measured in the fluorimeter (VICTOR^®^ Nivo^TM^ Plate Reader, PerkinElmer Inc, Waltham, MA, USA). The enzyme activity was determined according to a calibration curve constructed using 4MU, and normalized to 1 mg/mL of protein.

#### 4.5.2. Other Enzymatic Assays for Additional Lysosomal Enzymes

Other lysosomal enzyme activities were also measured in control (WT) and MPS II-derived SHEDs, namely, beta-galactosidase (GLB1, E.C. 3.2.1.23, the enzyme deficient in either GM1-gangliosidosis or MPS IVB), beta-glucuronidase (GUSB, E.C. 3.2.1.31, the enzyme deficient in MPS VII), hexosaminidase A (HEXA, E.C. 3.2.1.52, the enzyme deficient in GM2-gangliosidosis), alpha-N-acetyl-glucosaminidase (NAGLU, E.C. 3.2.1.50, the enzyme deficient in MPS IIIB) and alpha-galactosidase (GLA, E.C. 3.2.1.22, the enzyme deficient in Fabry disease). All measurements relied on the breakdown of specific 4MU substrates, except for that of arylsulfatase B (ARSB, E.C. 3.1.6.12), which relied on the use of an artificial chromogenic, substrate 4-nitrocatechol sulfate [62,63]. The enzyme activity was determined according to a calibration curve done with 4MU, and normalized to 1 mg/mL of protein.

#### 4.5.3. Glycosaminoglycans Quantification by LC-MS/MS

GAGs were quantified by simultaneous analysis of DS and HS in WT and MPS II-derived SHED homogenates, by LC-MS/MS, after butanolysis reaction, according to the method recently described by Forni and co-workers [55,64]. While initially described to perform HS and DS analysis in urine samples, this method was adapted to quantify the same compounds in cell homogenates. Briefly, cell homogenates were prepared by sonication, and their protein concentration determined with the same method described in Section 4.5.1. Each individual cell homogenate was divided into two different sample tubes, one for HS and another for DS. Samples were then dried under a stream of nitrogen and 75 µL of 3N HCl in N-butanol added to each vial. For HS measurements, samples were incubated for 60 min at 90 °C. For DS measurements, on the other hand, samples were heated for 25 min at 65 °C. After those incubations, samples were cooled back to room temperature for 10 min and dried under a stream of nitrogen. 100 µL of a 30:70 water/acetonitrile (*v*/*v*) solution were then added to each HS tube, and 250 µL to each DS tube and briefly vortexed. Then, the DS samples were combined with their respective HS counterparts and vortexed again. Finally, dimers derived from butanolysis reactions were chromatographed on HPLC using a gradient of acetonitrile and water (LC column: Gemini^®^ 3 µm C6-Phenyl 110 Å, 100 × 2 mm, from Phenomenex, Torrance, CA, USA) and detected on a triple quadrupole mass spectrometer (API4000 QTRAP from Sciex, Redwood City, CA, USA). Samples were quantified by interpolation from the calibration curve (prepared to cover a concentration range from 0.39 to 50 µg/mL for HS and from 1.56 to 100 µg/mL for DS using seven different levels) and reported in mg/mL. Then, HS and DS were normalized to protein concentration and finally reported as mg/mg protein.

#### 4.5.4. LAMP1 Immunostaining

LAMP1 immunostaining was performed as previously reported in Encarnação et al. [65]. Briefly, WT and MPS II-derived SHEDs were seeded (approximately 25,000 cells/well) in 8-well chamber slides (Nunc, Roskilde, Denmark), fixed with 4% PFA/PBS for 30 min, quenched with 0.05 M NH_4_Cl, permeabilized in ice-cold methanol for 10 min, and blocked with 5% BSA (Sigma-Aldrich, St. Louis, MO, USA), according to standard procedures. Coverslips were washed three times in PBS and once in water, mounted (Fluoroshield^TM^ with DAPI, Sigma-Aldrich, St. Louis, MO, USA) and examined by fluorescence microscopy (Leica TCS-SPE confocal microscope; Leica, Wetzlar, Germany). Spectral detection adjusted for the emission of DAPI and Alexa Fluor™ fluorochromes using the 405-, 488-, and 546-laser lines, respectively. Digital images were analyzed using ImageJ version 2.0.0. Details on the antibodies used and their respective dilutions are listed in Table 3.

#### 4.5.5. Lysosphingolipid Quantification by LC-MS/MS

Simultaneous quantification of sphingoid bases (lysosphingolipids) were quantified by LC-MS/MS as previously reported [57]. Cell pellets were lysed by sonication on ice, and a lysolactosylceramide internal standard was added to each sample. Protein concentration for each sample was determined with the same method described in Section 4.5.1. Then, sphingoid bases were extracted from cell lysates by a modified Bligh and Dyer extraction with acidic buffer [66]. Briefly, a mixture of chloroform:methanol was added to the samples, stirred an subsequently centrifuged for protein precipitation. Then, chloroform:methanol:water was used to extract the sphingoid bases, which were eluted in the upper phase after subsequent rounds of stirring and centrifugation. Eluted sphingoid bases were dried under a stream of nitrogen and reconstituted in methanol for LC-MS/MS detection on a triple-quadrupole mass spectrometer (API4000 QTRAP from Sciex, Redwood City, CA, USA). Samples were quantified by interpolation from the calibration curve and reported in pmolg/mL, and subsequently normalized to protein concentration (pmolg/mg protein).

### 4.6. Multi-Lineage Differentiation Protocol

The multi-lineage differentiation capacity of the established SHED cell lines was assessed with a straightforward protocol to promote SHEDs differentiation into the three germ layers.

Briefly, control and patient-derived SHEDs (px5–px7) were plated onto 24-well plates (8000 viable cells/cm^2^) and cultured in standard media until reaching a confluency of 70–80% of culture surface. The Human Pluripotent Stem Cell Functional Identification Kit (R&D Systems, Minneapolis, MN, USA) was then used to differentiate into endoderm and ectoderm, according to the manufacturer’s instructions. For mesoderm differentiation, however, a slight alternation to the original protocol was performed, by adding the CHIR99021 supplement (Stemcell Technologies, Vancouver, BC, Canada), a WNT pathway activator, to the Differentiation Base Media Supplement (50X), which is part of the kit Stem Cell Functional Identification, as previously reported [38]. For the immunocytochemistry, antibodies against Otx2, Brachyury, and Sox17 were used as markers for ectoderm, mesoderm and endoderm, respectively. Briefly, seeded SHEDs were fixed in 4% paraformaldehyde (Sigma-Aldrich, St. Louis, MO, USA) for 10 min, incubated with PBST with 1% BSA (Sigma-Aldrich, St. Louis, MO, USA) for 30 min and stained by standard immunofluorescence procedures. Cells were analyzed on DM400 M fluorescence microscope (Leica, Wetzlar, Germany). Antibodies are listed in Table 3.

### 4.7. Statistical Analysis

Statistical analysis was performed using GraphPad PrismVR (version 9, GraphPad Software, La Jolla, CA, USA). Results are presented as means ± standard error of the mean (SEM). Comparisons between groups were performed by one-way analysis of variance followed by Tukey’s multiple comparisons test. Differences were considered statistically significant when *p* ≤ 0.05. Significant results between groups are presented using the symbol (*). Significance results are also indicated according to *p* values with one, two, three or four of the symbols (*) corresponding to 0.01 < *p* ≤ 0.05, 0.001 < *p* ≤ 0.01, 0.0001 < *p* ≤ 0.001 and *p* ≤ 0.0001, respectively.

## 5. Conclusions

Here, we present our results on the establishment and characterization of two MPS II patient-derived stem cell lines from deciduous teeth. Quite remarkably, hallmark LSD features, namely, the presence of storage (GAGs, in this particular case) and of an abnormal LAMP1 staining pattern, are already evident in these cells. This is particularly relevant, since those features were not reported by other groups in MPS II-derived iPSCs [19]. Instead, iPSCs seem to need further differentiation in order to show the storage phenotype, which usually becomes evident only in NPCs or neural stem cells [19,20]. Altogether, this is a completely new observation in the field, and we believe it holds potential to set a new trend for investigating not only the subcellular pathology of virtually any LSD but also any gene expression changes that may occur in these pathologies. It may also allow for reliable genotype–phenotype correlations, as soon as significant samples from the same disorder are collected and their derived cell lines established.

Overall, this method relies on a non-invasive, cost-effective approach that can be set as a routine in any lab with standard cell culture conditions. By incredibly diminishing the costs associated with the establishment of a pluripotent cell line (which would otherwise rely on expensive, laboriouso and time-consuming iPSC generation and characterization protocols), this protocol may allow a significant number of laboratories to establish their first LSD-derived stem cell lines. The fact that some of the major LSD pathological hallmarks are already evident in the SHED state, even before any differentiation protocol is attempted, further validates the modeling potential of these dental-derived MSC cultures for this challenging group of disorders.

Having documented the major primary LSD pathological hallmarks in the established cell lines, our goal is now to increase the catalogue of MPS diseases with immortalized/established SHED cell lines, as well as that of available phenotypes for the same disease. Then, we will perform comparative analyses on a number of additional parameters, including secondary storage of lipids [not only by lysosphingolipid biomarker quantification as performed here, but also by analyzing GM2 and GM3 gangliosides, bis(monoacylglycero)phosphate and total cholesterol levels], and pathophysiological assessments [e.g., evaluations of the apoptosis rate or endoplasmic reticulum (ER) stress levels].

## Figures and Tables

**Table 1:** Relative expression levels of several markers, including *CD105*, *CD73*, *CD90* and *CD166* (MSC markers), *Sox-2*, *Oct-3/4*, and *Nanog* (pluripotency markers), *CD117*, *CD34*, *MHC I* and *MHC II*, in SHEDs from patients and controls, and also iPSCs derived from Fabry fibroblasts. Differences were based on qRT-PCR results and calculated using the standard ΔCt method, with *GAPDH* and *ACTB* as housekeeping genes.

Marker	SHEDs (2.01)	SHEDs (2.02)	iPSCs (INSAi002-A)
*CD105*	***	***	***
*CD73*	***	***	***
*CD90*	***	***	***
*CD166*	***	***	
*MHC I*	***	***	
*Sox-2*	*	*	**
*Oct-3/4*	*	*	***
*Nanog*	*	*	**
*CD117*	**	**	
*CD34*	*	*	*
*MHC II*	*	**	**

*** Strong expression; ** moderate expression; * weak expression.

**Table 2:** Summary of the most relevant clinical data from each MPS II patient, including age of diagnosis, symptoms and age of starting treatment.

Case	Age of Diagnosis (Years)	Symptoms	Age of Starting Treatment (Years)
2.01	3	Coarse facies, stiff joints, etc. Post-natal macroglossia Mild psychomotor development retardation; Interventricular communication (IVC) and patent ductus arteriosus (PDA); Moderate aortic insufficiency and left ventricular hypertrophy; Hydrocele; Chronic nasal obstruction without recurrent otitis or hearing deficit.	-
2.02	2	Inguinal hernias; Claw hands; Low stature; Hypertrichosis; Hepatomegaly; Cardiac involvement.	4

**Table 3:** Antibodies used in this study.

		Antibody	Dilution	Company Cat# and RRID
Primary Antibodies	Germ Layer Markers	Goat Anti-Human Brachyury Polyclonal Antibody, unconjugated (Mesoderm)	1:10	R&D Systems (Minneapolis, MN, USA) Cat# AF2085, RRID:AB_2200235
		Goat Anti-Human Sox17 Polyclonal Antibody, unconjugated (Endoderm)	1:10	R&D Systems Cat# AF1924, RRID:AB_355060
		Goat Anti-Human Otx2 Polyclonal Antibody, unconjugated (Ectoderm)	1:10	R&D Systems Cat# AF1979, RRID:AB_2157172
	Neural Stem Cell Markers	Mouse Anti-Human Nestin Monoclonal Antibody	1:49	Thermo Fisher Scientific (Waltham, MA, USA) Cat# MA1-110 (A24345)
		Rabbit Anti-Human PAX6	1:49	Thermo Fisher Scientific Cat# (A24340)
		Goat Anti-Human SOX1	1:49	Thermo Fisher Scientific Cat# (A24347)
		Rabbit Anti-Human SOX2	1:49	Thermo Fisher Scientific Cat# (A24339)
	LAMP1 Staining	Mouse Anti-Human LAMP-1 Monoclonal Antibody	1:200	Santa Cruz Biotechnology, Inc. (Dallas, TX, USA) Cat# sc-20011
Secondary Antibodies		Alexa Fluor 488 Donkey anti-Goat IgG (H + L) Cross-Adsorbed Secondary Antibody	1:200	Thermo Fisher Scientific Cat# A-11055, RRID:AB_2534102
		Alexa Fluor™ 488 Donkey Anti-Mouse; for use with anti-Nestin	1:249	Thermo Fisher Scientific Cat# (A24350)
		Alexa Fluor™ 488 Donkey Anti-Goat; for use with anti-SOX1	1:249	Thermo Fisher Scientific Cat# (A24349)
		Alexa Fluor™ 555 Donkey Anti-Rabbit; for use with anti-PAX6 or anti-SOX2	1:249	Thermo Fisher Scientific Cat# (A24342)
		Alexa Fluor™ 594 Donkey Anti-Rabbit; for use with anti-PAX6 or anti-SOX2	1:249	Thermo Fisher Scientific Cat# (A24343)

## Data Availability

Data are contained within the article and Supplementary Materials.

## Supplementary Materials

The supporting information can be downloaded at https://www.mdpi.com/article/10.3390/ijms25063546/s1.
