# Supplementary material for: Modeling Lysosomal Storage Disorders in an Innovative Way: Establishment and Characterization of Stem Cell Lines from Human Exfoliated Deciduous Teeth of Mucopolysaccharidosis Type II Patients
Source: Int J Mol Sci. 2024 Mar 21;25(6):3546. doi: 10.3390/ijms25063546 (PMC10970756; doi:10.3390/ijms25063546)
Supplement: Supplementary file 1 [file ijms-25-03546-s001.zip › ijms-2823442-supplementary.pdf]

Table S1: Absolute ΔCt values obtained by qRT-PCR of several markers, including *CD105*, *CD73*, *CD90* and *CD166* (MSC markers), *Sox-2*, *Oct-3/4*, and *Nanog* (pluripotency markers), *CD117*, *CD34*, *MHC I* and *MHC II*, in SHEDs from patients and controls, and also iPSCs derived from Fabry fibroblasts. Differences were calculated using *GAPDH* and *ACTB* as housekeeping genes.

Marker	SHEDs (2.01)	SHEDs (2.02)	iPSCs (INSAi002-A)
CD45	5.42	4.95	7.26
CD73	5.01	6.45	5.87
CD90	3.96	3.69	1.73
CD166	4.80	6.19	--
MHC I	2.82	3.07	--
Sox-2	19.71	20.56	11.10
Oct-3/4	12.92	12.60	8.02
Nanog	15.84	16.07	8.88
CD117	11.49	12.46	--
CD34	15.65	18.49	12.93
MHC II	13.09	9.63	10.32

Table S2: Medium values obtained by simultaneous quantification of sphingoid bases by LC-MS/MS: globotriaosylsphingosine (Lyso-Gb3) glucosylsphingosine (Glc-Sph), phosphorylcho-line-sphingosine (Lyso-SM) and phosphorylcho-line-sphingosine 509 (Lyso-SM-509). Two independent samples from each cell line were analysed in parallel for each biomarker.

**Biomarker**	**Glc-Sph**	**Lyso-Gb3**	**Lyso-SM**	**Lyso-SM-509**
**Associated Disorder**	Gaucher	Fabry	Niemann-Pick type AB	Niemann-Pick type C
Control SHEDs (n=2)	0.6	1.3	0.7	1.4
MPS II SHEDs (n=2)	0.3	1.5	1.2*	0.4

* For Lyso-SM, a 2-fold increase was observed in MPS II SHEDs *vs* controls, but that difference is not significant when compared with the routine measurements for Niemann-Pick type AB patients.

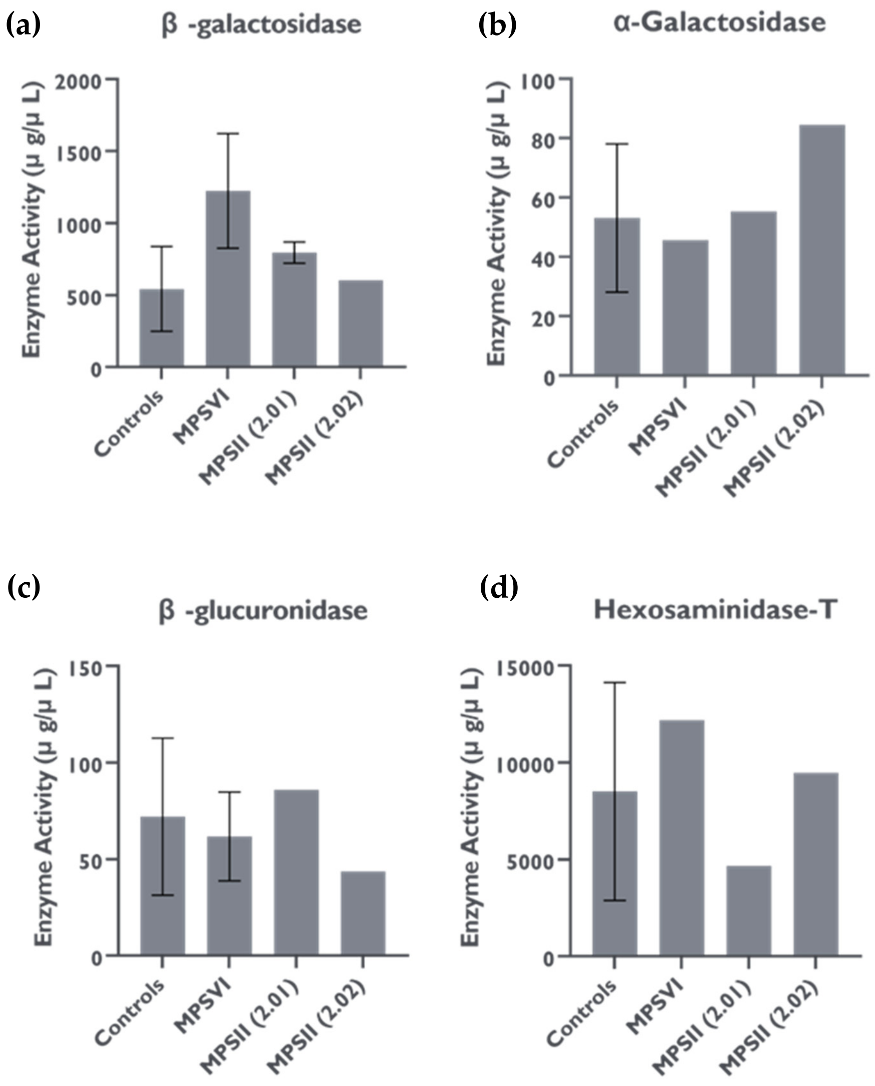

Figure S1. Enzyme activity measurements for additional lysosomal enzymes not directly involved in MPS II pathology are not significantly different level between control and MPS SHEDs. **(a)** beta-galactosidase (GLB1, E.C. 3.2.1.23, the enzyme deficient in either GM1-gangliosidosis or MPS IVB); **(b)** alpha-galactosidase (GLA, E.C. 3.2.1.22, the enzyme deficient in Fabry Disease); **(c)** beta-glucuronidase (GUSB, E.C. 3.2.1.31, the enzyme deficient in MPS VII) and **(d)** total hexosaminidase [includes hexosaminidase A (HEXA, E.C. 3.2.1.52, the enzyme deficient in GM2-gangliosidosis)]. Values for a similar MPS VI cell line, which we have also established in house, are also included as controls.

Quem somos:

Somos um grupo de Investigação & Desenvolvimento em **Doenças Lisossomais de Sobrecarga (DLS)**, sediado no Instituto Nacional de Saúde Doutor Ricardo Jorge, no Porto.

Liderado pela Doutora Sandra Alves*, o nosso grupo tem-se dedicado há mais de uma década ao estudo de uma série de DLS. As Mucopolissacaridoses, no entanto, têm sido um dos alvos preferenciais do nosso estudo.

No início da década passada dedicámo-nos a estudar as bases moleculares da doença, estabelecendo diagnósticos e analisando amostras de doentes Portugueses, de modo a perceber, ao nível do DNA/RNA qual o defeito genético de cada um. Mais tarde, decidimos usar esse conhecimento para tentar desenhar abordagens terapêuticas (também elas de base molecular) para tentar ultrapassar os defeitos moleculares que tínhamos identificado.

É no seguimento desses estudos, de resultados muito promissores, que surge este projecto, para o qual estamos a recrutar colaboradores.

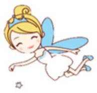

Por isso, estamos a pedir a colaboração de famílias com crianças com MPS que nos queiram ceder um dentinho.

Se está interessado(a) em colaborar connosco, não hesite em contactar-nos!

Contactos:

Maria Francisca Coutinho (francisca.coutinho@insa.min-saude.pt)

Sandra Alves* (sandra.alves@insa.min-saude.pt)

Pedido de Voluntários:

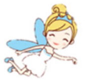

Projecto

A Fada dos Dentes 2020:

*um método para estabelecer **linhas celuleres neuronais***

a partir de dentes de leite de doentes com Mucopolissacaridoses

[1] Unidade de Investigação e Desenvolvimento, Departamento de Genética Humana, Instituto Nacional de Saúde Doutor Ricardo Jorge, Porto, Portugal

[2] Centro de Estudos de Ciência Animal , CECA-ICETA, Universidade do Porto, Porto, Portugal

Este é um projecto **inovador**, cujo objectivo é utilizar dentes de leite (caninos ou incisivos) de doentes com Mucopolissacaridoses (MPS) para gerar neurónios.

Esses neurónios serão posteriormente utilizados como modelo celular para testar abordagens terapêuticas inovadoras e tentar compreender o seu impacto ao nível do cérebro – um dos órgãos afectados nestes doentes e, para o qual ainda não há tratamento.

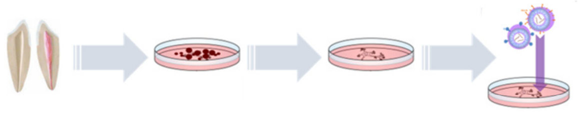

Para participar, basta ceder-nos um canino ou incisivo, logo que ele caia!

(ver detalhes na página seguinte)

Agradecimentos

Fontes de Financiamento:

Fundação para a Ciência e a Tecnologia
EXPL/BTM-SAL/0659/2021

Sanfilippo Children's Foundation – Funding research, finding gems
Incubator grant 2019DGH1656/SCF2019I&D

Bolsa SPDM de apoio à investigação Dr. Aguinaldo Cabral
2019DGH1629/SPDM2018I&D
Sociedade Portuguesa de Doenças Metabólicas

Apoio:

Little Drops of Water

Um projecto

Instituto Nacional de Saúde Doutor Ricardo Jorge

República Portuguesa – Saúde; SNS Serviço Nacional de Saúde; Instituto Nacional de Saúde Doutor Ricardo Jorge

Perguntas frequentes (FAQs)

Qual é o problema que estamos a tentar resolver?

A necessidade de **modelos celulares** adequados para testar o potencial de uma série de **drogas inovadoras**, cujo objectivo é actuar ao nível dos **sintomas neuronais**, presentes em muitas MPS.

É verdade que temos resultados promissores em células de pele de doentes (fibroblastos) mas o ideal será testar as drogas directamente nos seus tecidos/órgãos-alvo. Logo, nada melhor do que testá-las directamente em **neurónios**!

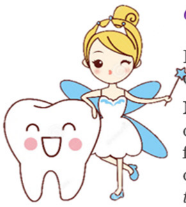

Qual é a vantagem de usar dentes de leite?

É uma amostra **não invasiva** e totalmente **isenta de dor** para o doente!

Nada de biópsias dolorosas! Os dentinhos caem de forma natural. Tudo o que a família tem de fazer é recolhê-los, colocar num líquido próprio que vamos preparar (chamado *meio de transporte*), e enviá-los para o nosso laboratório.

É difícil recolher/guardar os dentes? Precisam de ter algum cuidado especial?

Nem por isso mas verdade é que, para conseguirmos fazer o nosso trabalho no laboratório, há duas condições muit(íssim)o importantes:

- ✓ o dente deve ser colocado no *meio de transporte* no **máximo 20 min** depois de ter caído!
- ✓ tem de ser mandado para o laboratório nas **primeiras 48h**!

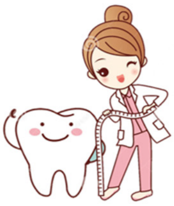

Quanto tempo demora até conseguirmos gerar neurónios a partir de dentes?

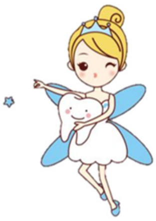

Embora tenhamos a ajuda de uma '*fada dos dentes*' muito especial, a verdade é que, no laboratório, nada surge '*a toque de varinha mágica*'; tudo leva o seu tempo.

No entanto, podemos dizer que, em condições ideais, o protocolo completo desde a chegada do dente ao laboratório até à obtenção de uma linha neuronal em cultura, leva no mínimo 6 a 8 semanas.

Mas…como é que a conseguimos gerar neurónios a partir de dentes, afinal?!

Claro que o processo laboratorial não é fácil de explicar, mas podemos dizer a coisa mais importante: os dentes de leite têm, no seu interior, uma categoria especial de células, chamadas '**células da polpa dentária**'. Algumas dessas células têm uma caraterística muito especial: são **células estaminais**.

A propriedade chave de todas as células estaminais é serem *indiferenciadas*. Isto significa que se conseguem replicar indefinidamente e substituir/renovar muitos tipos de células danificadas no organismo. Além disso, se soubermos dar-lhe o tratamento adequado no laboratório, conseguimos levá-las a dar origem a células especializadas, como é o caso dos **neurónios**!

Será garantido o estrito cumprimento de todos os requisitos legais, nomeadamente os relativos à proteção de dados, tais como a Diretiva comunitária de proteção de dados (Diretiva 95/46/EC do Parlamento Europeu e do Conselho de 24 de Outubro de 1995 sobre a proteção de indivíduos no que refere ao processamento de dados pessoais e à livre utilização dessa mesma informação) e as suas transposições nacionais. Neste contexto, será requerida uma autorização à Comissão Nacional de Proteção de Dados (CNPD) para recolha e processamento de dados pessoais, sempre que aplicável.

Quienes somos:

Somos un grupo portugués de Investigación y Desarrollo en **Enfermedades de Almacenamiento Lisosómico**, con sede en el Instituto Nacional de Salud Doctor Ricardo Jorge, en Oporto.

Nuestro grupo se ha dedicado durante más de una década al estudio de muchas de estas enfermedades. Sin embargo, las Mucopolisacaridosis (particularmente el tipo III) han sido uno de los objetivos preferidos de nuestro estudio.

Desde principios de la última década, estudiamos las bases moleculares de estas enfermedades, establecemos diagnósticos y analizamos muestras de pacientes, para comprender cuál es el defecto genético de cada uno a nivel de ADN/ARN. Recientemente hemos decidido utilizar este conocimiento para diseñar nuevos medicamentos (también de base molecular) para intentar superar los defectos moleculares que habíamos identificado. Para poder evaluar estos tratamientos, de los que tenemos resultados muy prometedores, hemos pensado en esta alternativa a las biopsias en este proyecto, para el que estamos reclutando voluntarios.

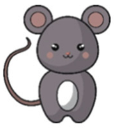

Para ello, pedimos la colaboración de familias con niños con MPS III que quieran donar un diente.

Si te interesa colaborar con nosotros, ¡no dude en contactarnos!

Contactos:

Maria Francisca Coutinho (francisca.coutinho@insa.min-saude.pt)

Sandra Alves* (sandra.alves@insa.min-saude.pt)

*Líder del grupo de investigación

Solicitud de voluntarios:

Proyecto

El ratoncito Pérez 2020*:

*"A Fada dos Dentes 2020" o "The 2020s Tooth Fairy" originalmente.

un proyecto para establecer líneas celulares neuronales de los dientes de leche de pacientes con Sanfilippo (MPS III)

[1] Unidade de Investigação e Desenvolvimento, Departamento de Genética Humana, Instituto Nacional de Saúde Doutor Ricardo Jorge, Porto, Portugal; [2] Centro de Estudos de Ciência Animal , CECA-ICETA, Universidade do Porto, Porto, Portugal; [3] Departamento de Biologia, Faculdade de Ciências da Universidade do Porto (FCUP), Portugal

"*El ratoncito Pérez de la década de 2020*" es un proyecto innovador, cuyo objetivo es utilizar dientes de leche (caninos o incisivos) de pacientes con síndrome de Sanfilippo (o Mucopolisacaridosis tipo III) para generar neuronas.

Estas neuronas se utilizarán más tarde como modelo celular para probar enfoques terapéuticos innovadores y tratar de comprender su impacto en el cerebro, el principal órgano afectado en los pacientes de Sanfilippo.

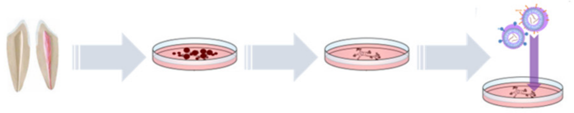

Para participar, ¡solo danos un canino o incisivo tan pronto como caiga!

(Ver los detalles en la próxima página)

Agradecimientos

Fuente de financiamiento:

Fundação para a Ciência e a Tecnologia EXPL/BTM-SAL/0659/2021

Sanfilippo Children's Foundation — Funding research, finding gems — Incubator grant 2019DGH1656/SCF2019I&D

Bolsa SPDM de apoio à investigação Dr. Aguinaldo Cabral 2019DGH1629/SPDM2018I&D — Sociedade Portuguesa de Doenças Metabólicas

Apoyo:

Little Drops of Water

Um proyecto

Instituto Nacional de Saúde Doutor Ricardo Jorge

República Portuguesa — Saúde; SNS Serviço Nacional de Saúde; Instituto Nacional de Saúde Doutor Ricardo Jorge

Preguntas frecuentes (FAQs)

¿Cuál es el problema que estamos intentando solucionar?

La necesidad de **modelos celulares** adecuados para probar el potencial de una serie de **fármacos innovadores**, cuyo objetivo es actuar a **nivel neuronal**, característico del síndrome de Sanfilippo (MPS III).

Tenemos resultados prometedores en células de la piel de los pacientes (fibroblastos), pero lo ideal sería probar los fármacos directamente en sus tejidos/órganos diana. Entonces, ¡nada mejor que probarlos directamente en **neuronas**!

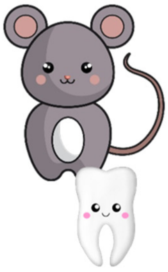

¿Cuál es la ventaja de usar dientes de leche?

¡Es una muestra **no invasiva** y totalmente **indolora** para el paciente!

¡Sin dolor ni biopsias! Los dientes se caen naturalmente. Lo único que tiene que hacer la familia es recogerlos, colocarlos en un líquido especial que vamos a preparar (llamado *medio de transporte*) y enviarlos a nuestro laboratorio.

¿Cuánto tiempo pasa antes de que podamos generar neuronas a partir de los dientes?

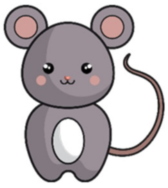

Aunque contamos con la ayuda de un *ratoncito Pérez*' muy especial, lo cierto es que, en el laboratorio todo lleva su tiempo.

Esperamos que, en condiciones ideales, el protocolo completo desde la llegada del diente al laboratorio hasta la obtención de una línea neuronal en cultivo, lleve de 6 a 8 semanas.

¿Es difícil recolectar/almacenar dientes? ¿Necesitas algún cuidado especial?

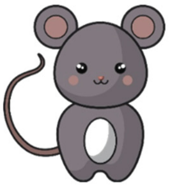

La verdad es que no, pero lo cierto es que, para poder hacer nuestro trabajo en el laboratorio, hay dos condiciones muy importantes:

- ✓ ¡El diente debe colocarse en el *medio de transporte* a **más tardar 20 minutos** después de que se haya caído!
- ✓ ¡Debe enviarse al laboratorio dentro de las **primeras 48 horas**!

Pero ... ¡¿cómo logramos generar neuronas a partir de los dientes?!

Aunque el proceso de laboratorio completo es largo de explicar, hay un dato importante: los dientes de leche tienen, en su interior, unas células con una característica muy especial: son células madre.

Esto significa que pueden replicarse indefinidamente y reemplazar muchos tipos de células dañadas en el cuerpo. Además, si sabemos darles el tratamiento adecuado en el laboratorio, ¡podemos hacer que den lugar a células especializadas, como las neuronas!

Será garantido o estrito cumprimento de todos os requisitos legais, nomeadamente os relativos à proteção de dados, tais como a Diretiva comunitária de proteção de dados (Diretiva 95/46/EC do Parlamento Europeu e do Conselho de 24 de Outubro de 1995 sobre a proteção de indivíduos no que refere ao processamento de dados pessoais e à livre utilização dessa mesma informação) e as suas transposições nacionais. Neste contexto, será requerida uma autorização à Comissão Nacional de Proteção de Dados (CNPD) para recolha e processamento de dados pessoais, sempre que aplicável.

Qui nous sommes::

Nous sommes un groupe de recherche et développement sur les **Maladies de Surcharge Lysosomales**, à l'Institut National de la santé Docteur Ricardo Jorge, à Porto (Portugal).

Notre groupe se consacre à l'étude des différents maladies lysosomales. Les mucopolysaccharidoses (en particulier le syndrome de Sanfilippo) ont cependant été l'une des cibles privilégiées de notre étude.

Au début de la dernière décennie, nous nous sommes consacrés à étudier les bases moléculaires de la maladie, à établir des diagnostics et à analyser des échantillons de patients portugais, afin de comprendre, au niveau de l'ADN/ARN, quel est le défaut génétique de chacun. Plus tard, nous avons décidé d'utiliser ces connaissances pour essayer des approches thérapeutiques (également à base moléculaire) pour tenter de surmonter les défauts moléculaires que nous avions identifiés.

C'est à la suite des résultats très prometteurs de ces études, que ce projet apparaît, dans lequel nous avons besoin de volontaires. Et pour ça, nous demandons la collaboration des familles avec des enfants atteints de MPS III qui veulent donner une dent.

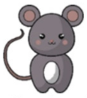

Si vous êtes intéressé à collaborer avec nous, n'hésitez pas à nous contacter!

Contacts:

Maria Francisca Coutinho (francisca.coutinho@insa.min-saude.pt)

Sandra Alves* (sandra.alves@insa.min-saude.pt)

*Chef de groupe de Recherche

Reconnaissance

Source de financement:

Fundação para a Ciência e a Tecnologia

EXPL/BTM-SAL/0659/2021

Sanfilippo Children's Foundation — Incubator grant 2019DGH1656/SCF2019I&D

Bolsa SPDM de apoio à investigação Dr. Aguinaldo Cabral 2019DGH1629/SPDM2018I&D — Sociedade Portuguesa de Doenças Metabólicas

Support:

Little Drops Water

Demande de volontaires:

Projet

La Petite Souris 2020*:

*"A Fada dos Dentes 2020" ou alors "The 2020s Tooth Fairy" dans la version originale

un projet pour l'établissement de lignées cellulaires neuronales des dents de lait de patients atteints du syndrome de Sanfilippo (MPS III)

[1] Unidade de Investigação e Desenvolvimento, Departamento de Genética Humana, Instituto Nacional de Saúde Doutor Ricardo Jorge, Porto, Portugal; [2] Centro de Estudos de Ciência Animal, CECA-ICETA, Universidade do Porto, Porto, Portugal; [3] Departamento de Biologia, Faculdade de Ciências da Universidade do Porto (FCUP), Portugal

«*La Petite Souris des années 2020*» est un projet **innovateur**, dont le but est d'utiliser les dents de lait (canines ou incisives) de patients atteints du syndrome de Sanfilippo (aussi appelé mucopolysaccharidose de type III) pour générer des neurones.

Ces neurones serviront plus tard de modèle cellulaire pour tester des approches thérapeutiques innovantes et tenter de comprendre leur impact sur le cerveau, le principal organe affecté dans cette maladie.

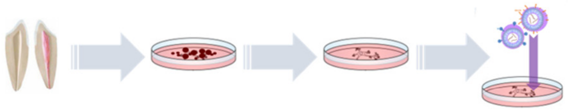

Pour participer, il suffit de nous donner une canine ou une incisive, juste après qu'il tombe!

(voir détails page suivante)

Un projet

Instituto Nacional de Saúde Doutor Ricardo Jorge

República Portuguesa — Saúde | SNS Serviço Nacional de Saúde | Instituto Nacional de Saúde Doutor Ricardo Jorge

Questions fréquentes (FAQs)

Quel est le problème que nous essayons de résoudre?

Le besoin de **modèles cellulaires** adéquats pour tester le potentiel d'une gamme de **médicaments innovants**, dont le but est d'agir au niveau des **symptômes neuronaux**, si caractéristiques du syndrome de Sanfilippo (MPS III).

Il est vrai que nous avons des résultats encourageants sur les cellules cutanées des patients (fibroblastes) mais l'idéal serait de tester les médicaments directement sur leurs tissus/organes cibles. Donc, rien de mieux que de les tester directement sur les **neurones**!

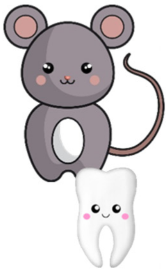

Quel est l'avantage d'utiliser des dents de lait?

C'est un échantillon **non invasif** et **totalement indolore** pour le patient!

Pas de biopsies! Les dents tombent naturellement. Ce qu'il faut, c'est que la famille attrape les dents, pour les placer dans un liquide que nous allons préparer (qu'on appelle moyen de transport), et les envoyer à notre laboratoire.

Combien de temps faut-il avant de pouvoir générer des neurones à partir des dents?

Bien que nous ayons l'aide d'une «*Petite Souris*» très spéciale, la vérité est que, dans le laboratoire tout prend son temps.

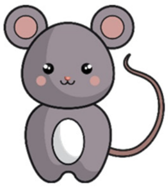

Cependant, on peut dire que, dans des conditions idéales, le protocole complet depuis l'arrivée de la dent au laboratoire jusqu'à l'obtention d'une lignée neuronale en culture, prend au minimum 6 à 8 semaines.

Est-il difficile de collecter/stocker les dents?
Avez-vous besoin de soins particuliers?

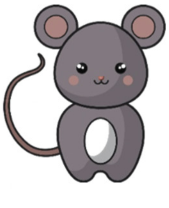

Pas vraiment, mais la vérité est que, pour pouvoir faire notre travail en laboratoire, il y a deux conditions très importantes:

- ✓ la dent doit être placée dans le moyen de transport **au plus tard 20 min** après qu'il est tombé!
- ✓ il doit être envoyé au laboratoire dans les **premières 48 heures**!

Mais… comment réussissons-nous à générer des neurones à partir des dents?!

Bien sûr, le processus de laboratoire n'est pas facile à expliquer, mais on peut dire le plus important: à l'intérieur des dents de lait, il y a une catégorie spéciale de cellules, les **cellules souches de la pulpe dentaire**.

La propriété clé de toutes les cellules souches est qu'elles sont indifférenciées. Cela signifie qu'ils peuvent se répliquer indéfiniment et remplacer/renouveler de nombreux types de cellules endommagées dans le corps. De plus, on donnant un traitement adéquat en laboratoire, on peut obtenir des cellules spécialisées, comme les **neurones**!

Será garantido o estrito cumprimento de todos os requisitos legais, nomeadamente os relativos à proteção de dados, tais como a Diretiva comunitária de proteção de dados (Diretiva 95/46/EC do Parlamento Europeu e do Conselho de 24 de Outubro de 1995 sobre a proteção de indivíduos no que refere ao processamento de dados pessoais e à livre utilização dessa mesma informação) e as suas transposições nacionais. Neste contexto, será requerida uma autorização à Comissão Nacional de Proteção de Dados (CNPD) para recolha e processamento de dados pessoais, sempre que aplicável.

Figure S2. Miniature versions of the flyers we have prepared to be distributed at the Portuguese hospitals/reference centres for LSD, drawing attention to our need for baby teeth. This call for volunteers was promoted under the designation "*A Fada dos Dentes 2020*" [Portuguese]/"The 2020s Tooth Fairy" [English]. Three different versions are available: in Portuguese, in Spanish and in French. For the Spanish and French versions, a slight adaptation was made to the project title to accommodate for cultural differences between the different target countries. Indeed, the folklore in those countries does not include a tooth fairy, as it does in Portugal or England, but a mouse: "*El Ratoncito Pérez*" [Spanish]/"*La Petite Souris*" [French]. Regardless of the language, all flyers included a brief summary of the project and its goals, major recommendations and frequently asked questions.
